# Engineering Mesoporous Structure in Amorphous Carbon Boosts Potassium Storage with High Initial Coulombic Efficiency

**DOI:** 10.1007/s40820-020-00481-7

**Published:** 2020-07-13

**Authors:** Ruiting Guo, Xiong Liu, Bo Wen, Fang Liu, Jiashen Meng, Peijie Wu, Jinsong Wu, Qi Li, Liqiang Mai

**Affiliations:** 1 State Key Laboratory of Advanced Technology for Materials Synthesis and Processing, Wuhan University of Technology, Wuhan, 430070 People’s Republic of China; 2 Foshan Xianhu Laboratory of the Advanced Energy Science and Technology Guangdong Laboratory, Xianhu Hydrogen Valley, Foshan, 528200 People’s Republic of China

**Keywords:** Potassium-ion battery, Mesopores engineering, Storage mechanism, Initial Coulombic efficiency

## Abstract

Amorphous carbon shows great potential as an anode material for high-performance potassium-ion batteries; however, its abundant defects or micropores generally capture K ions, thus resulting in high irreversible capacity with low initial Coulombic efficiency (ICE) and limited practical application. Herein, pore engineering via a facile self-etching strategy is applied to achieve mesoporous carbon (meso-C) nanowires with interconnected framework. Abundant and evenly distributed mesopores could provide short K^+^ pathways for its rapid diffusion. Compared to microporous carbon with highly disordered structure, the meso-C with Zn-catalyzed short-range ordered structure enables more K^+^ to reversibly intercalate into the graphitic layers. Consequently, the meso-C shows an increased capacity by ~ 100 mAh g^−1^ at 0.1 A g^−1^, and the capacity retention is 70.7% after 1000 cycles at 1 A g^−1^. Multiple in/ex situ characterizations reveal the reversible structural changes during the charging/discharging process. Particularly, benefiting from the mesoporous structure with reduced specific surface area by 31.5 times and less defects, the meso-C generates less irreversible capacity with high ICE up to 76.7%, one of the best reported values so far. This work provides a new perspective that mesopores engineering can effectively accelerate K^+^ diffusion and enhance K^+^ adsorption/intercalation storage.

**Electronic supplementary material:**

The online version of this article (10.1007/s40820-020-00481-7) contains supplementary material, which is available to authorized users.

## Highlights

- A facile self-etching strategy was used to obtain mesoporous carbon (meso-C) nanowires with zinc-catalyzed short-range ordered structure.
- Meso-C anode showed high initial Coulombic efficiency (76.7%) and excellent cycling stability (1000 cycles) for potassium-ion batteries.
- In/ex situ characterizations revealed the reversible structural changes, and the kinetic analyses revealed the rapid K^+^ diffusion in electrode.

**Electronic supplementary material:**

The online version of this article (10.1007/s40820-020-00481-7) contains supplementary material, which is available to authorized users.

## Graphical abstract

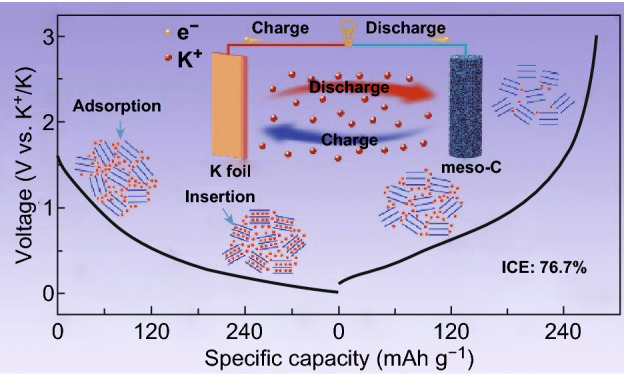

## Introduction

Potassium-ion batteries (PIBs) have attracted increased attention due to their potential to realize large-scale energy storage [1–3]. On the one hand, as the redox potential of K^+^/K (− 2.93 V vs. reversible hydrogen electrode) is close to that of Li^+^/Li (− 3.04 V) and lower than that of Na^+^/Na (− 2.71 V), PIBs are more likely to achieve high energy density [4]. On the other hand, potassium resources are abundant (17,000 ppm) in the Earth’s crust and cheap aluminum can be used as the current collector; thus, PIBs can be cost-effective [5]. Moreover, K^+^ has the smallest Stokes’ radius (3.6 Å) in propylene carbonate solvents when compared to those of Li^+^ (4.8 Å) and Na^+^ (4.6 Å), which leads to faster ion mobility in these electrolytes [6]. In spite of these advantages, scientific issues including the huge volume variation and sluggish ion transport in electrode materials caused by the much larger crystallographic radius of K^+^ (1.38 Å) than that of Li^+^ (0.76 Å) are still unresolved yet. For example, the commonly studied anode materials such as alloys Sn, Sb, P, or their alloys [7–9], sulfides [10] and selenides [11–13], suffered severe capacity decay problem although they could achieve high capacity.

Among traditional carbon materials, graphite anode has been applied in commercial lithium-ion batteries. However, the formation of KC_8_ causes a huge volume expansion of 61% during K-insertion process, which is much larger than that of LiC_6_ (10%) and thus leads to poor cycling life in PIBs [14, 15]. To resolve these issues, growing efforts focus on amorphous carbon (AC) or defects engineering on graphitic carbon [16–18]. AC possesses enlarged interlayer spacing and abundant defects, and the adsorption of K^+^ on defects contributes major capacity. Such physical process is faster and easier to be accomplished with negligible volume variation. Besides, the introduction of more defects was reported to promote surface-controlled energy storage by doping various heteroatoms (e.g., N, O, S and P) [19–21]. However, limited by such a surface-dependent storage mechanism, the working voltage was relatively wide, and the initial Coulombic efficiency (ICE) of AC anodes was usually below 60%. The high irreversible capacity and thus low ICE were mainly attributed to more irreversible consumption of electrolyte due to side reactions [22]. Recently, theoretical analysis confirmed that defect sites tended to capture metal ions due to their high migration barriers [23]. Experimental studies also indicated that AC with abundant defects or high porosity resulted in more irreversible capacity, and a high temperature treatment strategy was generally adopted to resolve the problem [24, 25]. Besides, reducing the specific surface area is also a commonly used method, but the difficulty is how to obtain high capacity, as the major capacity contribution originates from the surface sites. According to theoretical calculation results, planar graphitic regions in hard carbon contribute to the reversible metal ion storage, and curved pores endow metal ions with rapid diffusion, especially with the pore diameter of > 2 nm for K^+^ [26]. Thus, constructing mesoporous carbon with short-range ordered structure is expected to be an effective way, which could also provide additional space to buffer volume change [27, 28]. Hence, challenges exist in constructing well-distributed mesopores in amorphous carbon and understanding the structure-performance correlation.

Herein, the mesoporous carbon nanowires with short-range ordered structure were prepared for enhanced potassium storage by a simple self-etching strategy. The additive zinc ions could crosslink polyvinyl alcohol (PVA) and then form tiny ZnO nanoparticles during heat treatment, which are evenly dispersed in the pyrolytic carbon nanowires. The carbon-confined ZnO nanoparticles serve as templates and are in situ thermally reduced to metallic zinc by carbon and then zinc evaporates, resulting in the formation of homogeneous mesopores in the entire nanowires. Zinc also acts as the catalyst to generate the short-range ordered structure in mesoporous carbon (meso-C). The formed mesopores can not only accelerate K^+^ transport, but also promote K^+^ adsorption on the surface defects and further intercalation in the layered graphitic regions. Compared to microporous carbon (micro-C) nanowires formed by the pyrolysis of PVA nanowires, the meso-C shows greatly enhanced electrochemical properties and different potassium storage mechanism. Specifically, the ICE of meso-C can reach as high as 76.7%. Besides, its capacity is increased by ~ 100 mAh g^−1^ when compared to that of micro-C, exhibiting a high specific capacity of 278 mAh g^−1^ at 0.05 A g^−1^ and superior cycling stability with the capacity retention of 70.7% after 1000 cycles at 1 A g^−1^.

## Experimental Section

### Material Synthesis

Firstly, 3.0 g zinc acetate (Zn(Ac)_2_), 2.0 g low molecular weight (86–89% hydrolyzed, average M.W. 10,000–26,000), 1.6 g middle molecular weight (86–89% hydrolyzed, average M.W. 57,000–66,000), and 1.2 g high molecular weight PVA (87–89% hydrolyzed, average M.W. 88,000–97,000) were dissolved in 40 mL deionized water and then stirred slowly at 80 °C for 3 h to form a transparent, viscous and homogeneous electrospinning solution. The solution was then loaded in a 10-mL syringe with a 19-gauge blunt tip needle and electrospinning with a high voltage of 12 kV and a low voltage of − 2 kV. The distance between the nozzle and collector was 15 cm. The obtained nanowires were dried at 230–250 °C in air followed by annealed at 800 °C for 4 h in Ar with a heating rate of 4 °C min^−1^, and finally the meso-C nanowires were obtained. The precursor of micro-C nanowires was prepared without addition of zinc salt into the electrospinning solution, and the micro-C nanowires were obtained after the same heat-treatment condition.

### Material Characterization

Powder X-ray diffraction (XRD) analyses were performed on a Bruker D8 Advance X-ray diffractometer with Cu Kα radiation (*λ* = 1.5406 Å). Thermogravimetric and differential scanning calorimetry (TG/DSC) curves were recorded with Netzsch STA 449F3 simultaneous thermal analyzer. Scanning electron microscopy (SEM) images of samples were collected using a JEOL-7100F microscopy. Transmission electron microscopy (TEM) and high-resolution TEM (HRTEM), high-angle annular dark field scanning transmission electron microscopy (HAADF-STEM) images and selected-area electron diffraction (SAED) patterns were recorded using a JEM-2100F/Titan G260-300 transmission electron microscope working at 200 kV. Raman spectra were performed on Renishaw INVIA micro-Raman spectroscopy system. Brunauer–Emmett–Teller (BET) surface areas were measured using a Tristar II 3020 instrument by nitrogen adsorption at 77 K. In situ TEM characterizations were carried out by a Talos F200S. X-ray photoelectron spectroscopy (XPS) measurements were taken by an ESCALAB 250Xi instrument.

### Electrochemical Measurements

The coin cells (2016-type) were assembled in a UNIlab Plus glove box workstation filled with pure argon gas. The electrodes were fabricated by mixing active materials, acetylene black and carboxymethyl cellulose (CMC) sodium salt (mass ratio of 8:1:1), and then the slurry was spread onto the smooth Cu foil and dried for at least 10 h at 60 °C and finally punched to an electrode in a diameter of 10 mm with a mass loading of ~ 1 mg cm^−2^. K foil was separated from the working electrode using a glass microfiber filter (Whatman, Grade GF/D). The used electrolyte was 0.8 M KPF_6_ in ethylene carbon (EC)/diethyl carbonate (DEC) (1:1 in vol%). Galvanostatic charging/discharging tests, rate performance, cycling performance and the galvanostatic intermittent titration technique (GITT) were performed in the potential range from 0.01 to 3 V on the LAND CT2001A multichannel battery testing system. Cyclic voltammetry (CV) and electrochemical impedance spectroscopy (EIS) measurements were taken using CHI600E and Autolab PGSTAT 302 N electrochemical workstation.

## Results and Discussion

### Formation Mechanism of Meso-C

A two-step procedure was adopted to fabricate meso-C nanowires (see details in Experimental section). It includes the fabrication of Zn(Ac)_2_/PVA composite nanowires via electrospinning followed by controlled pyrolysis which is accompanied with thermal evaporation reactions. For comparison, electrospinning without the Zn(Ac)_2_ salt was also conducted, resulting in the micro-C nanowires.

**Fig. 1:**
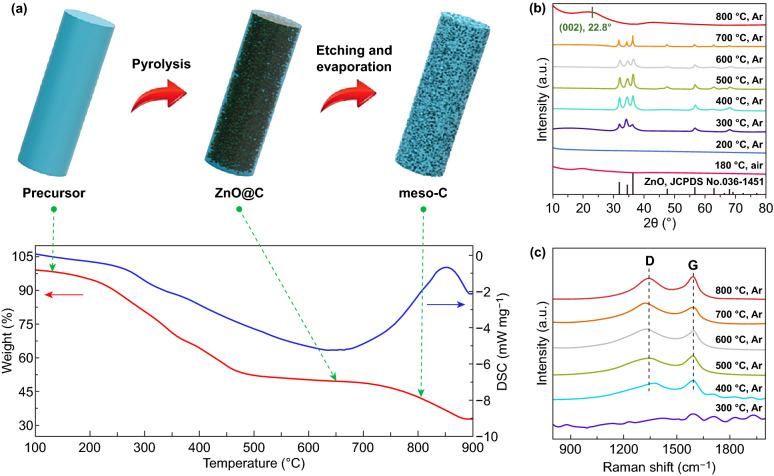
Formation mechanism of meso-C. **a** Schematic diagram for the formation of meso-C and the TG/DSC curves of Zn(Ac)_2_/PVA during heat treatment in Ar atmosphere. **b**, **c** Temperature-dependent ex situ XRD and ex situ Raman spectra during the formation processes of meso-C, respectively

Firstly, the formation mechanism of meso-C is analyzed in detail. Figure 1a depicts the TG/DSC curves of Zn(Ac)_2_/PVA in Ar atmosphere, with the schematic diagram of temperature-dependent microstructure/phase evolution. Initially, the Zn(Ac)_2_/PVA composite displays the solid nanowire structure with smooth surface as the TEM image shows (Fig. S1a). Due to the water-soluble property and low melting point (230–240 °C) of PVA, the composite was preheated in air at 230–250 °C to achieve dehydration and thermostabilization of PVA [29, 30]. Meanwhile, zinc ions are expected to crosslink PVA to form Zn–O bond with the hydroxyl groups [31]. After this process, the composite can effectively avoid deliquescence and maintain the durable nanowire structure. As shown in the immersion test, the preheated Zn(Ac)_2_/PVA remains unchanged microstructure, but the original one does not (Fig. S2). Next, ZnO is formed when the temperature raises up to 300 °C as determined by XRD patterns (Fig. 1b). The mass of sample decreases as the temperature increases, which is attributed to the decomposition of preheated Zn(Ac)_2_/PVA and gas release. From ex situ Raman results, the representative D-band (1350 cm^−1^) and G-band (1580 cm^−1^) appear when the pyrolysis temperature reaches 400 °C, indicating the formation of carbon at this stage. When further calcined at 600–800 °C, the carbonization of PVA is gradually completed with the calculated *I*_D_/*I*_G_ values of 1.09 at 600 °C, 1.05 at 700 °C and 0.95 at 800 °C, confirming the decreased structural disorder with the heat temperature increasing. The microscopic characterizations on the sample calcined at 700 °C without heat preservation (Fig. S1b, c) show that the carbon nanowires are embedded with a large amount of well-dispersed and ultrasmall ZnO nanoparticles. HRTEM analysis indicates the existence of ZnO with a size of ~ 8 nm and shows an interplanar distance of 0.26 nm, corresponding to the (002) plane of hexagonal ZnO (JCPDS No. 36-1451). When the temperature reaches 800 °C, ZnO could be thermally reduced to metallic Zn by carbon, and subsequently vaporized away [32], which is reflected by the disappeared XRD peaks of ZnO (Fig. 1b). Benefiting from the ZnO nanoparticles as the templates, thermal-induced zinc evaporation leads to the in situ formation of mesopores, which are evenly distributed throughout the whole nanowires.

Though the usual boiling point of Zn is 908 °C, the nano-Zn evaporation is supposed to happen at 800 °C from the above XRD results. XPS measurements were taken to further prove the thermal evaporation of nano-Zn at 800 °C. C 1*s* peak at 284.7 eV and O 1*s* peak at 532 eV in the survey spectrum for meso-C indicate the existence of C and O, respectively (Fig. S3a). No peaks can be observed in the high-resolution Zn 2*p* core-level XPS spectrum (Fig. S3b), indicating the absence of Zn. According to energy-dispersive X-ray spectra (EDX) mapping results in Fig. S4a, b, the signals of Zn element for both meso-C and micro-C samples are negligible, and the observed light spots may result from the deviation of electron microscope. Their corresponding EDX spectra (Fig. S4c, d) further indicate the inexistence of zinc in two samples. Table S1 lists the elemental analyses from EDX results, showing the > 99% content of C element and the undetected Zn. All the above results collectively demonstrate the complete removal of Zn in meso-C. In addition, the removal of Zn under heat treatment at 800 °C was also reported in previous reports [33, 34].

### Structural Characterization

**Fig. 2:**
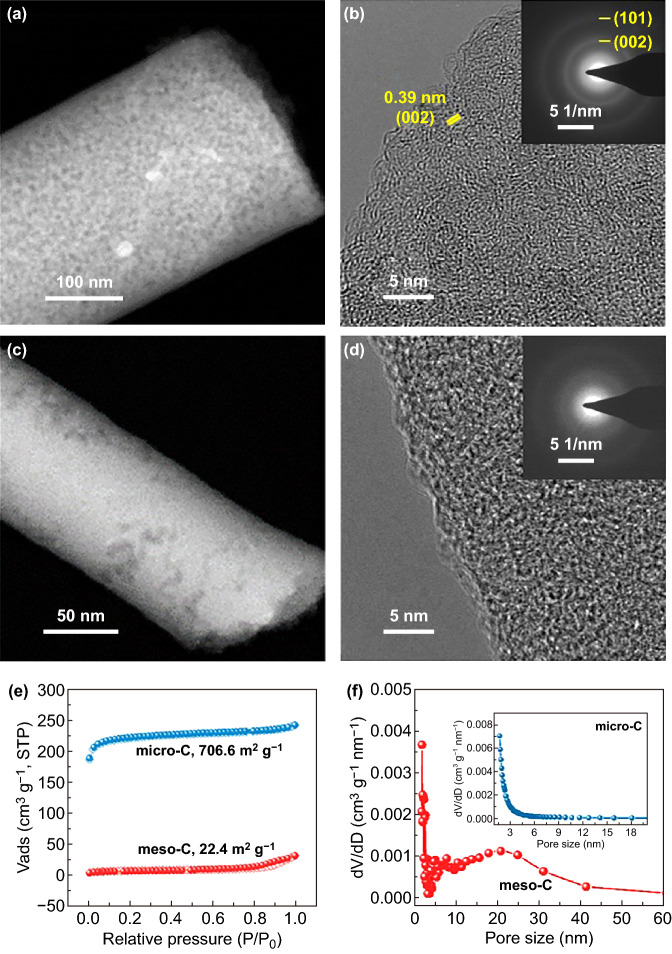
Structural characterizations. **a** HAADF-STEM and **b** HRTEM images of meso-C. Inset: the corresponding SAED pattern. **c** HAADF-STEM and **d** HRTEM images of micro-C. Inset: the corresponding SAED pattern. **e** Nitrogen adsorption–desorption isotherms and **f** pore size distribution curves of meso-C and micro-C

The microstructures of meso-C and micro-C are further characterized and compared in detail. SEM images of meso-C and micro-C and the corresponding statistical graphs of diameter distribution are displayed in Fig. S5. The meso-C nanowires are straight with a diameter of ~ 250 nm, while the micro-C displays twisty nanowire structure with a smaller diameter of ~ 200 nm. This may be attributed to the addition of zinc salts which leads to more rigid precursor nanowires due to its crosslinking with PVA. XRD pattern of meso-C (Fig. S6a) shows a broad peak at 22.8° with an average interlayer distance (*d*_002_) of 0.39 nm, while the same peak of micro-C is located at a lower angle with a larger interlayer distance of 0.41 nm. The in-plane graphitic crystallite size (*L*_a_) and the crystallite size along the *c*-axis (*L*_c_) were also determined by the Scherrer equation [35]. The *L*_a_/*L*_c_ values of meso-C and micro-C samples are 3.22/1.28 and 3.09/1.17 nm, respectively, further suggesting the higher graphitization degree of meso-C. The typical HAADF-STEM image of meso-C (Fig. 2a) confirms that the mesopores exist on the outer surface and the interior of the whole nanowire. The meso-C shows the short-range ordered graphited layers with a distinguishable spacing of 0.39 nm (Fig. 2b), and such distance is favorable for K^+^ diffusion [26]. However, the micro-C displays a solid structure without obvious porous structure from HAADF-STEM image (Fig. 2c). Meanwhile, it is a highly disordered structure of micro-C as shown in Fig. 2d. Compared to micro-C, the SAED pattern of meso-C with brighter and sharper diffraction ring suggests its higher graphitization degree (inserts in Fig. 2b, d). This is further verified by Raman results (Fig. S6b). Two peaks located at 1324 and 1591 cm^−1^ correspond to the D- and G-band of carbon, and the intensity ratio (*I*_D_/*I*_G_) between the D- and G-bands is generally used to define the degree of graphitization of carbon [36, 37]. Here the calculated I_D_/I_G_ values are 1.16 for micro-C and 0.95 for meso-C, confirming the higher degree of graphitization of meso-C, which may be attributed to the catalysis effect of Zn [38].

Nitrogen adsorption/desorption isotherm experiments were carried out to further characterize the pore structure (Fig. 2e). The type IV isotherms with H3-type hysteresis loop for meso-C indicate its mesoporous structure. Differently, the micro-C shows type I isotherms, and the high isotherm in a low-pressure region suggests its high microporosity [39]. The specific BET surface area of meso-C is 22.4 m^2^ g^−1^, much smaller than that of micro-C (706.6 m^2^ g^−1^). The pore diameter of mesopores for meso-C is mainly distributed in 5–30 nm; however, its distribution for micro-C is basically < 2 nm (Fig. 2f). Besides, pore size distribution curve of meso-C also suggests the existence of a small proportion of micropores, which is inevitable from the decomposition of PVA. In the end, the smaller specific surface area for meso-C originates from the introduction of mesopores and thus greatly reduced micropores.

### Electrochemical Performance

**Fig. 3:**
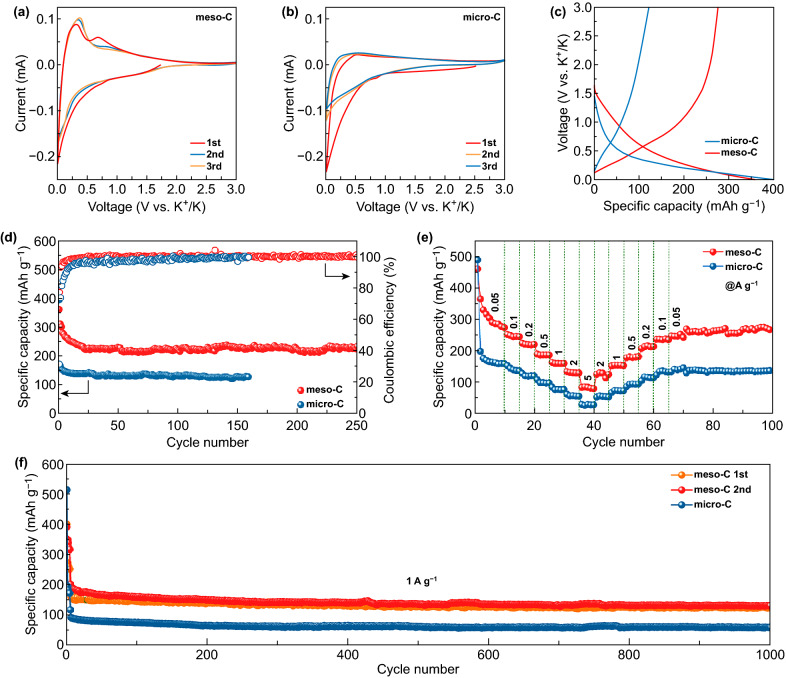
K-storage performance. **a**, **b** CV curves of meso-C and micro-C at 0.1 mV s^−1^ in 0.01–3.0 V. **c** First charging/discharging profiles. **d** Cycling performance at 0.1 A g^−1^ and **e** rate performance. **f** Long-term cycling performance at 1 A g^−1^ after initial 5 cycles at 0.1 A g^−1^

The electrochemical performance of meso-C as PIBs anode was firstly evaluated by CV measurements in a voltage window of 0.01–3.0 V (vs. K^+^/K). As shown in Fig. 3a, an extremely weak cathodic peak located at ~ 0.7 V only appears in the 1st cycle of meso-C, which is attributed to the irreversible decomposition of electrolyte to form solid-electrolyte interphase (SEI) [40]. The sharp cathodic peak at 0.01 V and the wide cathodic peak at 0.15 V could be attributed to intercalation of K ions to form K-C compound [41]. The anodic peaks at 0.3 and 0.7 V in the 1st cycle represent the extraction of K ions [42], and they shift to 0.375 and 0.88 V in the 2nd and 3rd cycles, respectively, which suggests an irreversible transition of the structure probably happens during the 1st cycle [27]. For micro-C, the small cathodic peak at 0.8 V is assigned to SEI formation (Fig. 3b). It is worth noting that there also exists a sharp cathodic peak at 0.01 V in 1st cycle CV of micro-C, and it becomes wider and weaker in the following cycles, indicating an irreversible potassium storage happens in 1st cycle. Particularly, the cathodic peak at 0.01 V of meso-C is maintained in the next two cycles, suggesting much more capacity contribution of reversible K^+^ intercalation reaction.

Figure 3c shows the charging/discharging curves in the first cycle at 0.1 A g^−1^. The calculated ICE for meso-C is 76.7%, which is much higher than that of micro-C (31.1%) and most reported carbon-based anodes (Table S2). Besides, the 1st cycle discharging curve can be divided into two regions, which includes the slope region (0.4–1.5 V) and the plateau region (0.01–0.4 V). For micro-C, compared with the following two cycles of discharge curves shown in Fig. S7b, the irreversible capacity in the 1st cycle is mainly generated in plateau region. The plateau region almost disappears in the next two cycles, suggesting an irreversible potassium storage, and such a result is consistent with the CV results. Besides, there are two slight plateaus in the charging curve of meso-C, while the charging curve of micro-C approximates a straight line. Such a phenomenon also suggests the difference in K-storage mechanism between these two samples, which will be discussed in detail later.

Long-term cycling performance was firstly evaluated at a small current density of 0.1 A g^−1^ (Fig. 3d). For meso-C, the specific capacity gradually reduces to ~ 230 mAh g^−1^ and the Coulombic efficiency (CE) goes up to 99% in the first 20 cycles and then remains stable. However, the CE of micro-C goes up to 99% after 120 cycles, which may be due to its higher specific surface area and thus needs longer time to form stable SEI layer [43]. Besides, meso-C still delivers a high discharge capacity of 231 mAh g^−1^ after 250 cycles, showing its excellent cycling stability, whereas the reversible capacity of micro-C at 0.1 A g^−1^ is ~ 130 mAh g^−1^, much smaller than that of meso-C. Rate performances for the two samples were also measured and compared (Fig. 3e). When tested at different current densities of 0.05, 0.1, 0.2, 0.5, 1, and 2 A g^−1^, the meso-C delivers the discharging capacities of 278, 240, 215, 184, 157, and 129 mAh g^−1^, respectively. However, the micro-C only delivers much lower capacities of 161, 138, 118, 96, 75, and 55 mAh g^−1^, respectively. After deep charging and discharging, when back to 0.05 A g^−1^, the meso-C displays a remained high capacity of 270 mAh g^−1^. Furthermore, the meso-C also shows a repeatable outstanding long cycling stability at 1 A g^−1^ (meso-C 1st and meso-C 2nd), and a high capacity of 133 mAh g^−1^ even after 1000 cycles with a capacity retention of 70.7% can be achieved (Fig. 3f). Figure S8 shows the TEM images of the electrodes after long-term cycling at 1 A g^−1^, where the meso-C displays the well-remained mesoporous nanowire structure, but the structure of micro-C nanowires exhibits slightly destroyed, confirming that the mesoporous structure is beneficial for accommodating the volume change and maintaining the structural stability. To sum up, the meso-C anode displays superior overall performances, and particularly, its specific capacity and ICE exhibit a significant improvement when compared to micro-C. This suggests the effectiveness of mesoporous engineering in AC anodes toward high-performance PIBs.

### Structural Evolution and Kinetics Analysis

**Fig. 4:**
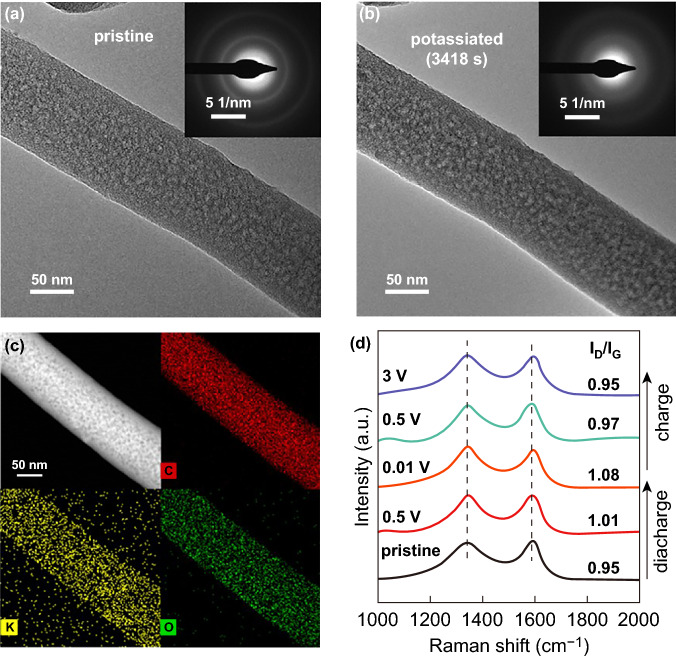
Structural evolution during potassiation and depotassiation. In situ TEM images and SAED patterns of **a** pristine and **b** potassiated meso-C nanowires. **c** HAADF-STEM image and the corresponding EDX mappings of potassiated meso-C nanowire. **d** Ex situ Raman spectra of meso-C

In situ TEM measurements were taken to study the structural evolution of meso-C during the potassiation process. The diameter of nanowire displays a negligible change (Fig. 4a, b). EDX mappings show the even distribution of C, K and O elements, indicating the successful insertion of K ions into the meso-C structure (Fig. 4c). The existence of abundant mesopores is responsible to buffer the volume change during the potassiation process. From the corresponding SAED pattern (insets in Fig. 4a, b), the two diffraction rings corresponding to the (002) and (101) planes are sharp and clear before potassiation and become quite diffused after potassiation. It indicates that the insertion of K^+^ not only results in slight expansion of the carbon platelets, but also leads to increased structural disordering. Such a phenomenon is also observed from ex situ SAED pattern of meso-C anode after discharging to 0.01 V (Fig. S9a). Furthermore, the diffraction ring becomes clear and sharp after depotassiation (Fig. S9b), indicating the reversibility of structural changes. Ex situ Raman spectra were also collected to further confirm the structure changes (Fig. 4d). The *I*_D_/*I*_G_ values increase from 0.95 to 1.08 when discharging to 0.01 V and then decrease to 0.95 when charging to 3 V, revealing the reversible changes in structure. This is consistent with the above in/ex situ microscopy results, and these results are also similar to the previous reported works [18, 44].

**Fig. 5:**
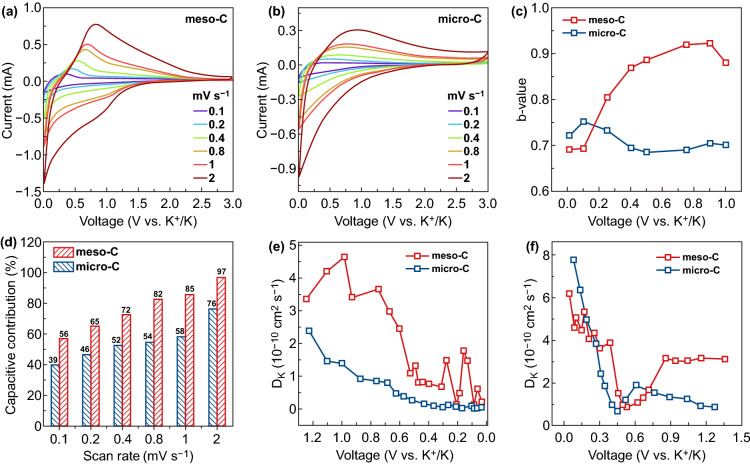
Kinetics analysis of K-storage. CV curves at various scan rates of **a** meso-C and **b** micro-C. **c** Calculated *b* values for meso-C and micro-C for cathodic sweep. **d** Contribution of the surface-controlled process in meso-C and micro-C at different scan rates. K-ion diffusion coefficient as a function of the state of **e** the discharging process and **f** the charging process

To investigate the kinetics of electrode materials, CV measurements at different scan rates (0.1–2 mV s^−1^) of meso-C and micro-C were taken (Fig. 5a, b). According to Eq. 1 [45]:

1 $$i = av^{b}$$

where *i* is current, *ν* is scan rate, both *a* and *b* are adjustable parameters. By plotting log (*i*) against log (*ν*), the values of *b* can be obtained from the slope of fitted curves. Traditional cation insertion is a diffusion-controlled process, thus the *b* value is expected to be close to 0.5. However, it would be close to 1 for a surface-controlled process. Here, the *b* values at 0.01–1 V for meso-C and micro-C for cathodic sweep are calculated (Fig. 5c). For micro-C, *b* values just fluctuate ~ 0.7 without obvious decrease or rise, while those for meso-C show a hill-top trend as the voltage decreases. From 1 to 0.4 V, the *b* values for meso-C are higher than 0.85, indicating the capacity at this stage is mainly capacitive. The *b* value becomes smallest at below 0.2 V, and as the intercalation of K^+^ has been proved to happen at low potential. Meanwhile, the decrease of *b* values with decreased potential indicates the active insertion reactions at this stage. Such results suggest the more active capacitive effect at a high potential and insertion reaction at a low potential for meso-C when compared to that of micro-C. The current response $i$ at a potential *V* can be divided into surface-dominated capacitive process ($k_{1} v$) and the diffusion-controlled intercalation process ($k_{2} v^{1/2}$) according to Eq. 2 [46]:

2 $$i\left( V \right) = k_{1} v + k_{2} v^{1/2}$$

$v$ is the scan rate. The $k_{1}$ values at varied potentials were firstly determined by the linear relationship of $i/v^{1/2}$ and $v^{1/2}$, and then the surface-dominated fraction ($k_{1} v$) can be further determined. The red region and blue region in Fig. S10a, b represent the surface-dominated fraction, suggesting a surface-dominated contribution of 72% for meso-C anode, which is higher than that of micro-C (52%). The ratios for both meso-C and micro-C increase as the scan rates increase (Fig. 5d), showing the importance of capacitive effects at high scan rates.

The different potassium storage processes are supposed to be related to structural differences of meso-C and micro-C. On the one hand, the amorphous carbon can provide surface defect sites for the adsorption of K^+^. Meanwhile, the short-range ordered regions in meso-C also provide more K^+^ insertion/extraction sites. On the other hand, the connected mesopores in meso-C provide short diffusion pathways for K^+^ to access the inner part of meso-C nanowires quickly. Figure S11a shows the EIS results fitted with the equivalent circuit model (Fig. S11b) for fresh cell at open circuit voltage (OCV). The obviously higher slope at the low-frequency sloping region indicates the faster ion diffusion feature of meso-C. The charge-transfer resistance (*R*_ct_) values of meso-C and micro-C are similar, further suggesting that the improved ion diffusion property plays an important role in enhancing the electrochemical performances of meso-C.

To further understand the kinetic processes, GITT was used to determine the K^+^ diffusion coefficients *D*_*k*_. Figure S12a shows the potential curves with the discharging time with a pulse current of 30 mA g^−1^ for 10 min and relaxation for 30 min in 0.01–3 V. The higher slope of meso-C in the slope region and the smaller overpotentials (the potential change during each relaxation period) indicate its better kinetic property [19]. The potential response of charging process was also determined by GITT measurement (Fig. S12b). The results show that less potassium is released for micro-C, even though which could store potassium with a similar amount in the 1st cycle compared to meso-C, suggesting a significant amount of potassium ions are trapped in micro-C. In the GITT curve of meso-C, there are two obvious plateau at around 0.2 and 0.6 V, further suggesting the extraction of K^+^ from graphited layers. The *D*_*k*_ values are calculated using the equation in Electronic supplementary material, and Fig. S13 shows the regional potential response with time. Figure 5e shows the calculated *D*_*k*_ values during the discharging process. The higher *D*_*k*_ of meso-C than micro-C indicates a much faster K-ion diffusion in meso-C, especially at the slope region, which could be attributed to the superiority of mesoporous structure. In addition, the diffusion behaviors are supposed to be closely related to the electrochemical process and the corresponding structural changes [18]. For the two samples, the *D*_*k*_ values at below 0.5 V are lower than that at high voltage region, because K ions have to overcome the repulsive charge gradient from surface to the interior turbostratic regions [19]. Figure 5f shows the *D*_*k*_ of meso-C during charging process. The expanded interlayer space after potassiation process could gradually recover with the extraction of K^+^. At this stage, the diffusion of K^+^ becomes sluggish, and thus the diffusion coefficients decrease until 0.5 V.

## Conclusions

In summary, we have successfully constructed mesoporous carbon nanowires by a facile self-etching strategy. The in situ pyrolysis and etching of zinc source contribute to the formation of homogeneous mesopores with Zn-catalyzed short-range ordered structure. Compared to micro-C with highly disordered structure, the meso-C shows an increased capacity by ~ 100 mAh g^−1^ when used as the PIBs anode, which exhibits a high specific capacity of 278 mAh g^−1^ at 0.05 A g^−1^, and could maintain 230 mAh g^−1^ after 250 cycles at 0.1 A g^−1^. Besides, meso-C also shows outstanding rate performance (129 mAh g^−1^ at 2 A g^−1^) and excellent cycling stability (1000 cycles at 1 A g^−1^). More importantly, benefiting from mesoporous structure with reduced specific surface area by 31.5 times, the ICE of meso-C can reach as high as 76.7%. Overall, our work offers a facile way to obtain carbon nanowires with interconnected mesoporous and short-range ordered structure, which greatly facilitate the K^+^ diffusion and provide more potassiation sites, thus leading to more active and reversible adsorption/intercalation of K^+^. This work is expected to provide a reference that mesoporous structure engineering in amorphous carbon is effective for high-performance PIBs with high ICE.

## Electronic supplementary material

Below is the link to the electronic supplementary material.

Supplementary material 1 (PDF 913 kb)
