# Supplementary material for: Engineering Mesoporous Structure in Amorphous Carbon Boosts Potassium Storage with High Initial Coulombic Efficiency
Source: Nanomicro Lett. 2020 Jul 13;12:148. doi: 10.1007/s40820-020-00481-7 (PMC7770926; doi:10.1007/s40820-020-00481-7)
Supplement: Supplementary file 1 — Supplementary material 1 (PDF 913 kb) [file 40820_2020_481_MOESM1_ESM.pdf]

Supporting Information for

Engineering Mesoporous Structure in Amorphous Carbon Boosts Potassium Storage with High Initial Coulombic Efficiency

Ruiting Guo[1], Xiong Liu[1], Bo Wen[1], Fang Liu[1], Jiashen Meng[1], Peijie Wu[1], Jinsong Wu[1], Qi Li[1,*], Liqiang Mai[1,2,*]

[1]State Key Laboratory of Advanced Technology for Materials Synthesis and Processing, Wuhan University of Technology, Wuhan 430070, People's Republic of China

[2]Foshan Xianhu Laboratory of the Advanced Energy Science and Technology Guangdong Laboratory, Xianhu hydrogen Valley, Foshan 528200, People's Republic of China

*Corresponding authors. E-mail: qi.li@whut.edu.cn (Qi Li); mlq518@whut.edu.cn (Liqiang Mai)

S1 Diffusion Coefficients Calculation

The K^+ diffusion coefficients were calculated by using the equation based on Fick's second law [S1]:

$$D = \frac{4}{\pi\tau}\left(\frac{n_B V_M}{S}\right)^2 \left(\frac{\Delta E_S}{\Delta E_\tau}\right)^2$$

where D is the K^+ diffusion coefficient, τ is the current pulse time (s), n_B is the amount of substance, V_M is the molar volume of the active material, and S is the area of the electrodes. ΔE_S is the potential difference of two adjacent steady-states and ΔE_τ is the potential change due to the pulse current.

S2 Supplementary Figures and Tables

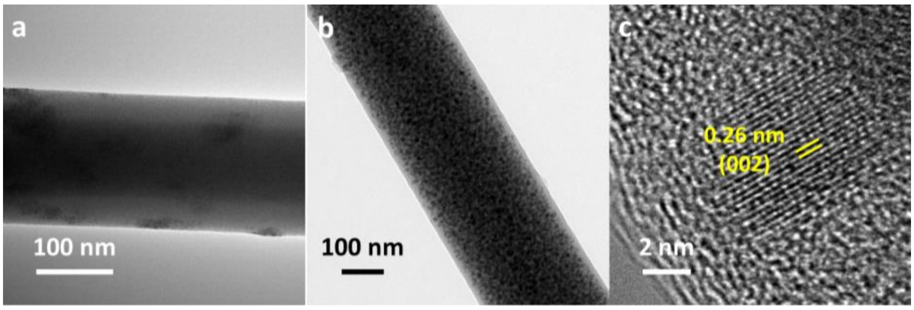

Fig. S1 (**a**) TEM image of preheated $Zn(Ac)_2$/PVA. (**b, c**) TEM and HRTEM images of ZnO@C

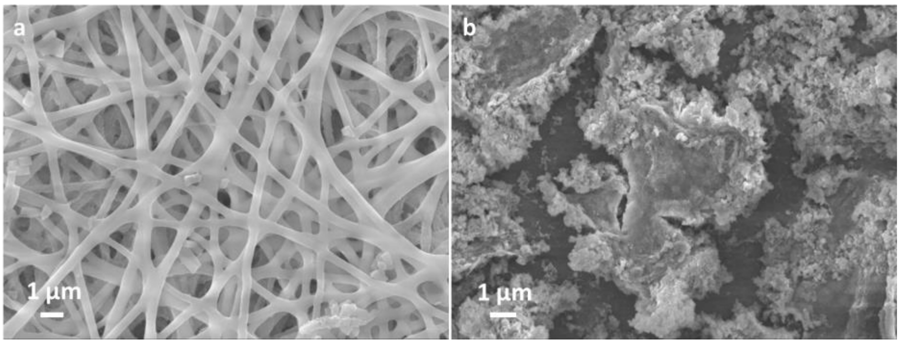

Fig. S2 SEM images of (**a**) preheated $Zn(Ac)_2$/PVA and (**b**) untreated $Zn(Ac)_2$/PVA after immersing in deionized water for 24 h

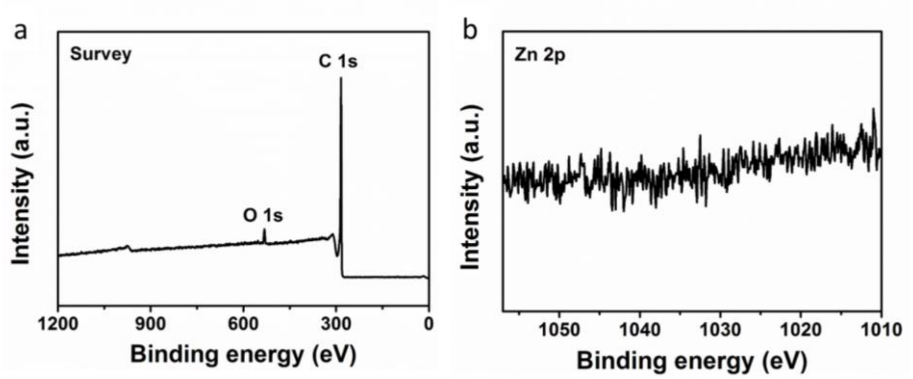

Fig. S3 (**a**) XPS survey spectrum and (**b**) high-resolution Zn 2p XPS spectrum for meso-C sample

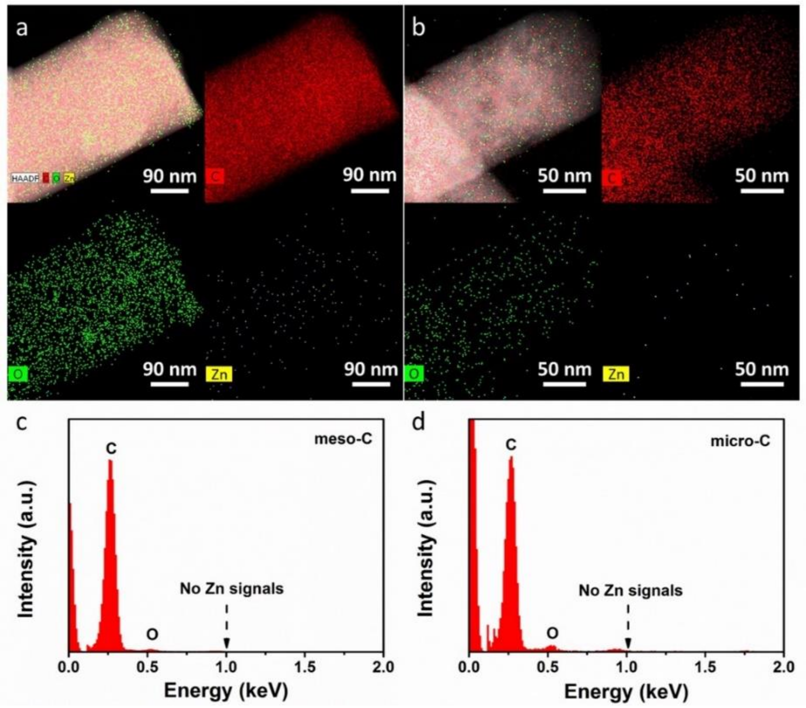

Fig. S4 STEM-EDX mappings of (**a**) meso-C and (**b**) micro-C, and corresponding EDX spectra of (**c**) meso-C and (**d**) micro-C

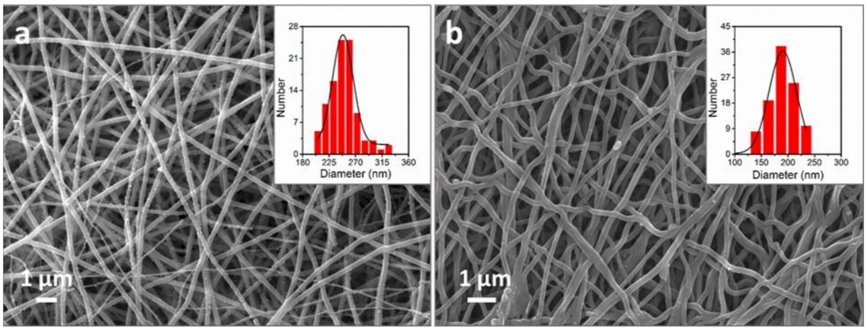

Fig. S5 SEM images and the corresponding diameter distributions (insets) of (**a**) meso-C and (**b**) micro-C nanowires

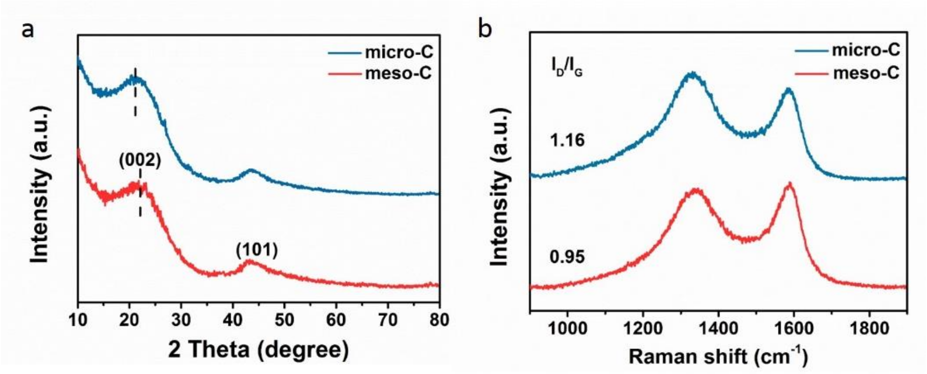

Fig. S6 (**a**) XRD patterns and (**b**) Raman spectra of meso-C and micro-C

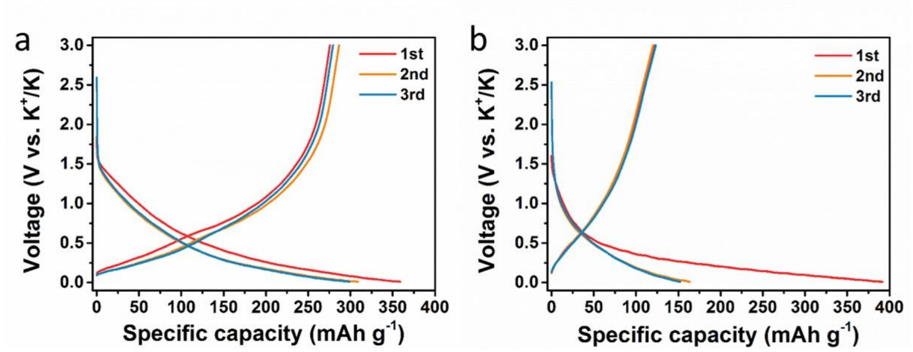

Fig. S7 Galvanostatic charging/discharging profiles of (**a**) meso-C and (**b**) micro-C tested at 0.1 A g^{-1}

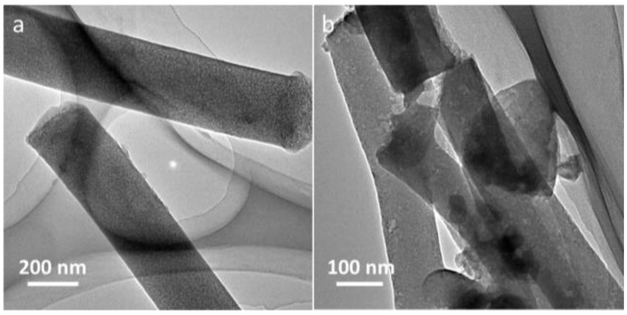

Fig. S8 TEM images of (**a)** meso-C and (**b**) micro-C after long-term cycling at 1 A g^{-1}

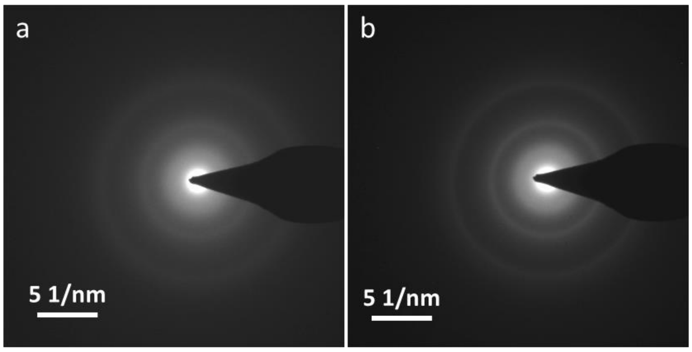

Fig. S9 Ex situ SAED patterns of meso-C (**a**) after discharging to 0.01 V and (**b**) charging to 3 V

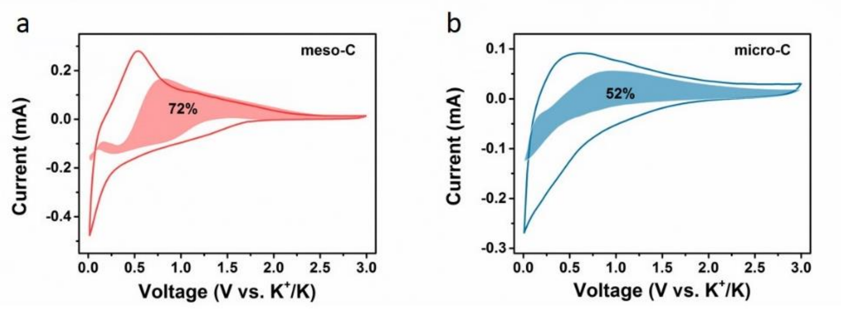

Fig. S10 Contribution of the surface process at 0.4 mV s^{-1} for (**a**) meso-C and (**b**) micro-C samples

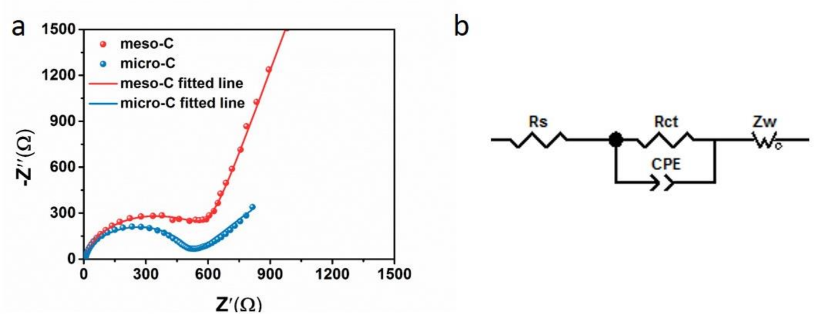

Fig. S11 (**a**) Electrochemical impedance spectroscopy curves for fresh cells at OCV (V vs. K^+/K). (**b**) The equivalent circuit used to fit the original data

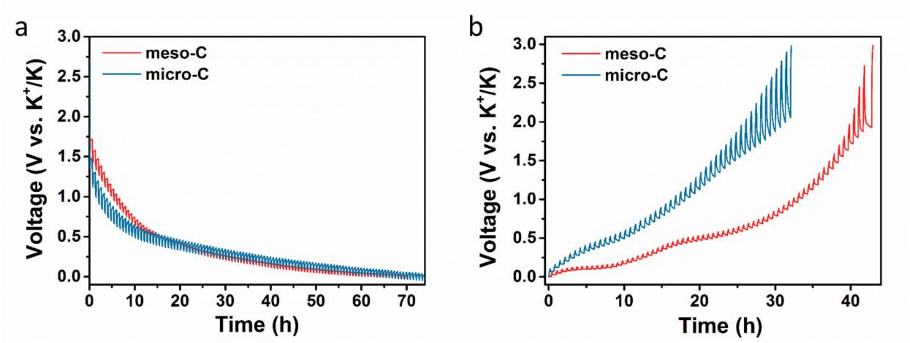

Fig. S12 (**a, b**) GITT curves of the discharging and charging processes, respectively

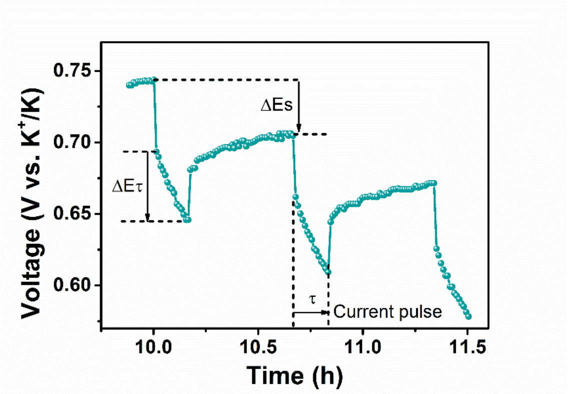

Fig. S13 Regional GITT potential response with time

Table S1 STEM-EDX mapping elemental atomic components of meso-C and micro-C samples

Samples	Element content (at.%)		
	C	O	Zn
meso-C	99.71	0.29	0.00
micro-C	99.02	0.89	0.00

Table S2 K-storage performance comparison of meso-C anode in this work with other reported carbonaceous anodes

Samples	Current density (mA g^{-1})	Capacity after cycling (mAh g^{-1})	Cycle number	ICE (%)	References
Hollow interconnected neuron-like carbon	140	250	150	72.1	[S2]
Hard-soft composite carbon	279	200	200	67	[S3]
S doped RGO-600	50	361	50	65	[S4]
Graphite	93	255	2000	64	[S5]
Ordered mesoporous carbon	50	257.4	100	63.6	[S6]
Sulfur-grafted hollow carbon spheres	200	300	250	51.4	[S7]
NCNFs	25	248	100	49	[S8]
SMCF@CNTs	279	193	300	48	[S9]
N/O dual-doped carbon network	50	303	50	47.1	[S10]
Graphitic carbon nanocage	55.8	212	100	40	[S11]
CNF-O	279	160	300	35	[S12]
NCNTs	50	254.7	300	24.4	[S13]
Porous carbon nanofiber	200	211	1200	24.1	[S14]
N-doped hollow carbon	200	225.4	1000	16	[S15]
Graphitic nanocarbon	200	189	200	15.5	[S16]
meso-C nanowires	100	231	250	76.7	This work

Supplemenary References

[S1] Z. L. Jian, Z. Y. Xing, C. Bommier, Z. F. Li, X. L. Ji, Hard carbon microspheres: potassium-ion anode versus sodium-ion anode. Adv. Energy Mater. **6**(3), 1501874 (2016). https://doi.org/10.1002/aenm.201670017

[S2] D. -S. Bin, X. -J. Lin, Y. -G. Sun, Y. -S. Xu, K. Zhang et al., Engineering hollow carbon architecture for high-performance K-ion battery anode. J. Am. Chem. Soc. **140**(23), 7127-7134 (2018). https://doi.org/10.1021/jacs.8b02178

[S3] Z. L. Jian, S. Hwang, Z. F. Li, A. S. Hernandez, X. F. Wang et al., Hard-soft composite carbon as a long-cycling and high-rate anode for potassium-ion batteries. Adv. Funct. Mater. **27**(26), 1700324 (2017). https://doi.org/10.1002/adfm.201700324

[S4] J. L. Li, W. Qin, J. P. Xie, H. Lei, Y. Q. Zhu et al., Sulphur-doped reduced graphene oxide sponges as high-performance free-standing anodes for K-ion storage. Nano Energy **53**, 415-424 (2018). https://doi.org/10.1016/j.nanoen.2018.08.075

[S5] L. Fan, R. F. Ma, Q. F. Zhang, X. X. Jia, B. G. Lu, Graphite anode for potassium ion battery with unprecedented performance. Angew. Chem. Int. Ed. **58**(31), 10500-10505 (2019). https://doi.org/10.1002/anie.201904258

[S6] W. Wang, J. H. Zhou, Z. P. Wang, L. Y. Zhao, P. H. Li et al., Short-range order in mesoporous carbon boosts potassium-ion battery performance. Adv. Energy Mater. **8**(5), 1701648 (2018). https://doi.org/10.1002/aenm.201701648

[S7] J. Ding, H. L. Zhang, H. Zhou, J. Feng, X. R. Zheng et al., Sulfur-grafted hollow carbon spheres for potassium-ion battery anodes. Adv. Mater. **31**(30), 1900429 (2019). https://doi.org/10.1002/adma.201900429

[S8] Y. Xu, C. L. Zhang, M. Zhou, Q. Fu, C. X. Zhao et al., Highly nitrogen doped carbon nanofibers with superior rate capability and cyclability for potassium ion batteries. Nat. Commun. **9**(1), 1720 (2018). https://doi.org/10.1038/s41467-018-04190-z

[S9] C. Shen, K. Yuan, T. Tian, M. H. Bai, J. -G. Wang et al., Flexible sub-micro carbon fiber@CNTs as anodes for potassium-ion batteries. ACS Appl. Mater. Interfaces **11**(5), 5015-5021 (2019). https://doi.org/10.1021/acsami.8b18834

[S10] J. F. Ruan, Y. H. Zhao, S. N. Luo, T. Yuan, J. H. Yang et al., Fast and stable potassium-ion storage achieved by in situ molecular self-assembling N/O dual-doped carbon network. Energy Storage Mater. **23**, 46-54 (2019). https://doi.org/10.1016/j.ensm.2019.05.037

[S11] B. Cao, Q. Zhang, H. Liu, B. Xu, S. L. Zhang et al., Graphitic carbon nanocage as a stable and high power anode for potassium-ion batteries. Adv. Energy Mater. **8**(25), 1801149 (2018). https://doi.org/10.1002/aenm.201801149

[S12] R. A. Adams, J. -M. Syu, Y. P. Zhao, C. -T. Lo, A. Varma, V. G. Pol, Binder-free N-and O-rich carbon nanofiber anodes for long cycle life K-ion batteries. ACS Appl. Mater. Interfaces **9**(21), 17872-17881 (2017). https://doi.org/10.1021/acsami.7b02476

[S13] P. X. Xiong, X. X. Zhao, Y. H. Xu, Nitrogen-doped carbon nanotubes derived from metal-organic frameworks for potassium-ion battery anodes. ChemSusChem **11**(1), 202-208 (2018). https://doi.org/10.1002/cssc.201701759

[S14] X. X. Zhao, P. X. Xiong, J. F. Meng, Y. Q. Liang, J. W. Wang, Y. H. Xu, High rate and long cycle life porous carbon nanofiber paper anodes for potassium-ion batteries. J. Mater. Chem. A **5**(36), 19237-19244 (2017). https://doi.org/10.1039/c7ta04264g

[S15] W. X. Yang, J. H. Zhou, S. Wang, W. Y. Zhang, Z. C. Wang et al., Freestanding film made by necklace-like N-doped hollow carbon with hierarchical pores for high-performance potassium-ion storage. Energy Environ. Sci. **12**(5), 1605-1612 (2019). https://doi.org/10.1039/c9ee00536f

[S16] W. L. Zhang, J. Ming, W. L. Zhao, X. C. Dong, M. N. Hedhili et al., Graphitic nanocarbon with engineered defects for high-performance potassium-ion battery anodes. Adv. Funct. Mater. **29**(35), 1903641 (2019). https://doi.org/10.1002/adfm.201903641
